# Summer Diseases of Children

**Published:** 1909-08

**Authors:** William Edward Fitch

**Affiliations:** New York City


For Texas Medical Journal.

### Summer Diseases of Children.

BY WILLIAM EDWARD FITCH, M. D., NEW YORK CITY.

The satisfactory treatment of bowel diseases of children are among the most difficult summer problems with which the physician has to deal. Usually the diagnosis is easy, and the results fairly satisfactory from proper medication, and yet the treatment of this class of diseases is unsatisfactory on account of the many outside influences, chief among which is the lack of intelligent cooperation on the part of the parent. Among the more prominent of these affections are summer diarrhoea, acute gastro-enteritis, iliocolitis, including the severer form—cholera infantum, to be the more representative types of these summer disturbances.

On careful investigation the etiology of these affections may be summed up as follows: Unhygienic surroundings, unwholesome water supply, uncleanliness, injudicious clothing and atmospheric changes, and allowing draughts of air to strike the child when being prepared for bed during the hot weather. According to the New York Milk Commission, the most prolific cause of "summer bowel disorders" is the free and indiscriminate use of unmodified cow's milk as an artificial food for infants and very young children. According to A. R. Reynolds, M. D., Health Commissioner of the city of Chicago, in a paper read before the California State Medical Association, April 20, 1909, and published in the Chicago *Medical Recorder,* May, 1909, he says:

"The conviction has been growing with me that cow's milk is a very much over-estimated article of diet for children. We know it has caused sickness again and again. It has carried to the child almost every known contagious disease, as well as the usual enteric diseases, because it is so easily infected, and the germ life multiplies so rapidly. It is a short-lived product at best, and every physician has seen bottle-fed babies raised without it. The more I think of it the more it seems to me that cow's milk as a diet for the child, instead of being a natural food, is quite an unnatural food.

"Nature provides that the milk go directly from the mother without change of temperature and sterile into the stomach of the offspring. All cow's milk is contaminated before it reaches the milk pail, even under the best conditions. The milk supply of cities is from twenty-four to forty-eight hours old before it reaches the child, and has become a very different article since leaving the udder of the cow.

"If physicians skilled in the artificial feeding of infants will give us a substitute, and I feel sure they can, the whole expensive and vexatious question of a city's milk supply will vanish like a dream.

"Nature demands that children with teeth should use them upon a solid diet. Indeed, mastication is necessary for the proper development of the teeth, and the digestive secretions of the mouth. Therefore, if a child is constantly urged to drink milk it is certain he can not eat and digest very much else.

"It is rather sweeping to recommend the elimination of cow's milk in cities from the diet of children under 5 years of age, but the still high sickness rate and the still higher mortality rate among the little ones is disconcerting, and I feel that the use of cow's milk is at the bottom of much of the trouble."

J. P. Crozier Griffith of Philadelphia, writing in the *Therapeutic Gazette*, July 15, 1906, in discussing the causative factors in "Summer Complaint," says:

"Bacteria as an etiological factor are generally recognized as the primary cause of these diarrhoeas. Milk is a particularly susceptible culture medium, and the germs multiply in it in hot weather with enormous rapidity. Even in winter the average dairy milk is loaded with micro-organisms, and in summer it is only by the greatest care that their growth can be restrained in the best of milk within limits which an infant can safely manage. That it is particularly in summer time that the excessive infantile mortality from diarrhoea develops, and that it is especially in bottle-fed infants that this occurs, prove the powerful influence which bacterial growth possesses. An additional proof is the great lessening of mortality among the poor to whom specially pure and properly modified milk is supplied."

Recognizing the danger of milk as a food for infants and children, the Canadian Medical Association and the Toronto Academy of Medicine have jointly had a bill introduced before the Legislature to create a "Milk Commission," whose duty will be to examine the milk supply of the larger cities of the Dominion. The *Canadian Journal of Medicine and Surgery,* in an editorial on this subject, says: "We predict that the Commission will discover that the milk supply of most of our larger cities is dirty, unfit for consumption, and is infected with disease germs. Many cows have tuberculosis, whose milk is being drunk by human beings, and beyond the possibility of doubt bovine tuberculosis is transmissible to human beings. Beyond doubt, typhoid fever, scarlet fever and most epidemics of diarrhoea and other intestinal diseases among children in the summer months are due directly to dirty milk."

The most common of this class is simple diarrhoea. Here we find the patient's temperature normal, the fever only a slight rise, and this probably due to disturbed digestion, over-heating, draughts of air, irritating food, or from fright. In the judicious handling of these cases search should always be made to determine the cause for therapeutic purposes. We should decide whether the condition is one of irritation per se or atony. The common fallacy of attributing all bowel disorders to teething is to be deprecated. This method has long since been excluded as unscientific. We all know that dentition may provoke distressing nervous phenomena, but it is absolute folly to attribute all diarrhoeal disturbances chiefly to teething.

The differential diagnosis of simple diarrhoea from the severer forms of summer complaint is not difficult. The light pea-green or dark green and greenish dejections of the irritative form is characteristic. For the sake of accurate diagnosis it would be wise to direct the nurse to fold up the diaper tight immediately upon removal, so as to exclude the air as far as possible, since oxidation rapidly takes place, and the normal color of the stool be changed to pea-green or a deep green color. The excoriation in the neighborhood of the anus is sometimes due to a strong alkali soap which has not been thoroughly rinsed from the diaper in the process of the laundering. It is well to remember this, since it may be attributed to other causes. If the dejections from the bowels are acrid they might produce a similar condition. The dejections from the bowels may be either accompanied with pain and straining or in the atonic form may be characterized by light-colored, profuse watery stools passed with little difficulty. There will be found undue relaxation, pale face, cool surface and a feeble circulation.

Another form of summer complaint frequently encountered is acute gastro-enteritis. This type usually follows a neglected or badly treated diarrhoea. It is an inflammatory disorder of grave import occurring during the second summer and frequently termed summer complaint. Three forms are met with in practice: Acute dyspeptic diarrhoea, acute gastro-enteritis and that graver form of this type of disease—cholera infantum.

The most frequent cause of summer complaint is atmospheric heat, especially during the hot midsummer nights passed in crowded, ill ventilated tenements under conditions inducing simple diarrhoea.

Second among the causative factors is weaning or change from the mother's milk to fresh cow's milk in one form or another. Indigestion is the first step in this catarrhal inflammation, for such is the picture occupying pathologically an intermediate ground between acute indigestion and ileo-colitis. The distressing symptoms accompanying the acute dyspeptic form alarms the close observing, watchful physician, who realizes what bowel disturbance of the second summer means and that it may suddenly take on a fatal character. In this condition the stools gradually increase in frequency, the child becomes more restless, with but little or no febrile symptoms. Increasing dejections become rapidly offensive, then gaseous, finally green and white speckled due to undigested casein, which acts as a foreign body, undergoes fermentation, produces increased inflammation and the gases evolved causes a painful distention of abdomen. Under such conditions all food should be stopped for twelve or twenty-four hours, a large dose of castor oil administered and only barley water given. This will at once change the character of the stools, the color will change to brownish and the putrid odor disappear. If allowed to continue the diarrhoea progresses and a tenacious mucus appears. The stools will become frothy and greenish, the patient will become feverish with loss of muscular tone and extreme emaciation follows.

In acute cases the attack is sudden, fever rapidly develops, while the evacuations are frequent and preceded by cramps and their voiding is attended with much tenesmus and tormonia. The belly is puffed, tender and tympanitic. Three is great thirst, the little patient will voraciously gulp at water or liquids, while vomiting of undigested food may result if the emesis be prolonged; it soon becomes mucous and bilious, and water so greedily gulped by the suffering child is immediately ejected from the stomach in a spouting stream. Convulsions may occur or the child may remain in a restless, febrile or heavy comatose state. As the warm weather continues these attacks may occur coincident with an unusually hot spell and may last for a week or ten days.

Cholera infantum (this is the most dreaded of all summer complaints of children and may prove fatal in a few hours). Ordinarily, slight diarrhoea, quickly followed by great thirst, incessant vomiting provoked easily upon drinking liquids and large exhaustive watery stools, ten to fifty a day, serve as landmarks to identify this most dreadful enemy of infancy and early childhood. Usually at first the stools contain small particles of fecal matter, quickly the evacuations change, becoming a colorless or nearly odorless serum. Prostration is profound and the body shows plainly the effect of the great and depressing drainage on the little patient's system. The deathly pallor, flabby muscles and skin, sunken eyes and fontanelles and retracted pinched facial expression are characteristic. The child in its agonizing nervous condition desires to change its position every few moments or to be carried in the arms of the mother or nurse. Stupor and convulsions are not uncommon preceding death. The temperature of these hopeless little patients may run as high as 110° F. in the axilla.

In the treatment of summer complaints each case requires special study and often taxes the skill of the most acute practitioner. However, as specific medication has proven, we need not, for therapeutic purposes, think of them as diseases, for our work is more successful when each condition is met by the indicated remedies as they arise.

The early nosological diagnosis is of the greatest importance, but the specific diagnosis points so unerringly to the best therapeutic progress. To be able to diagnose cholera infantum from other less virulous forms of bowel disorders gives the practitioner a clearer insight as to the future of the case so that he may give the parents a guarded prognosis.

It is important early in the case to impress upon the mother or nurse that no treatment will be successful without the utmost hygienic, painstaking care and prompt medication in these neglected cases. Absolute withdrawal of food is necessary in many instances and should be made peremptory, even though the child is fretting and crying—from hunger (as the parent thinks). A large percentage of these disorders could undoubtedly be prevented if those having the charge of children could understand or could realize the importance of fresh air, proper bathing and cleanliness in regard to clothing, regularity and moderation in feeding and the selection of a suitable artificial food for the child. The senseless custom of giving the baby sweetened teas, castoria, soothing syrups, paregoric or other sedative mixtures pave the way for many summer intestinal disorders by keeping the stomach and intestinal canal in a continual disturbed state. Insist that nothing—not even food—be given that has not been directed or sanctioned by the attending physician.

The necessity for scrupulous cleanliness in regards to nursing bottles and nipples should be impressed upon the mother or nurse. The careful selection of an artificial food, judicious bathing, with plain or alkalized water, and the immediate removal of soiled napkins to avoid excoriation are imperative. An abundance of pure, cool spring water, even though it be vomited (especially in cholera infantum, even when retched and vomited) should be given; while water is essential, it should always be given frequently and in small amounts. If vomiting does occur, its ejection from the stomach aids in washing out this organ and sometimes by overcoming gastric irritability, subdues vomiting. Ice is frequently enjoyed and should be crushed and tied in a gauze cloth to prevent the swallowing of large pieces. It is absolutely imperative that a reliable toilet powder, especially the borated products, should be copiously and liberally used, since they allay the distress caused from the acrid discharges. Excoriation from acrid stools may be prevented by anointing the neighborhood of the anus with olive oil, or, better still, mutton tallow; a heavy coating of the latter prevents the delicate skin from contact with the destructive secretions.

Healthy breast milk, from nature's fountain, the natural and only ideal food for babies, may be taken as the type of infant's food, and the nearer an artificial substance can be made to approach it in chemical composition and physical properties, the more perfect it is. Normal breast milk is a persistently alkaline fluid, having somewhat unusually disagreeable and very sweetish taste. It is bluish white in color, and thin and watery in consistence, and, according to Leeds, is composed of thirteen parts of solid and eighty-seven parts of water. (See analysis in text books.)

In seeking substitute for human milk, one naturally selects the milk of the lower animals, cow's milk being preferable, owing to its cheapness, and the fact that it is easily obtained. Cow's milk is richer looking—that is, whiter and more opaque than human milk, and is slightly acid in reaction, comparing the analysis of human milk with the following analysis of cow's milk. (See analysis of text books.) Thus we readily observe that the two fluids differ in specific gravity and reaction, and that cow's milk contains more nitrogenous material, but less fat and much less sugar than woman's milk. Undue attention has been given to the organic constituents of milk and too little to the mineral contents.

The relative proportions of casein and lactalbumin have been determined with sufficient accuracy to point out the most important of all the differences between the two secretions, which is, that the fraction of the total albuminoids in cow's milk which is coagulable by acids (casein) is far greater (perhaps four times) than the coagulable part (lactalbumin). In woman's milk, on the contrary, the reverse is true, and the non-coagulable part much exceeds (perhaps by more than twice) the coagulable portion. Taking weight for weight of each secretion, the coagulum of human milk is only one-fifth that of cow's milk.

This difference is readily determined by adding rennet to the two fluids. In cow's milk the casein is coagulated into large, firm masses, while with human milk a light, loose curd is formed. In the stomach the acid gastric juice has the same effect, producing, in the first instance, a coagulum must difficult to digest. In the other, one of vastly less bulk and readily attacked and easily broken down by the gastro-intestinal solvents.

To overcome these objections, a certain amount of cereals can be added to the cow's milk. The practice is in accord with the acknowledged teachings of such pediatrists as Drs. Jacobi. Chapin, Keller and Smith. The old contention that the infant had no distaste for the conversion of starch into sugar is no longer considered to be true. Heubner, of Berlin, has demonstrated that diastase can be found in the partotid gland of an infant two hours old, two weeks old and two months old. An infusion of parotid gland converts starch into sugar from the beginning of life. An infusion of pancreatic gland does not convert starch into sugar, and its diastasic power, even at the end of the year, is feeble.

An ideal modification of cow's milk as an artificial infant's food is a very knotty problem, but the author believes a carefully selected milk from pasture fed cows to which has been added cereals and maltose, will produce an artificial food which will meet the demands in the greatest number of cases. This disposes of the argument that the addition of cereals is the addition of foreign matter: trouble from the starch can be easily overcome by dextrinizing the gruel, and the mechanical separation of the particles of coagulum make a milk diluted in this way much more digestible than the milk diluted to the same extent with water.

Where the density of the coagulum is dependent upon the proportion of caseinogen, by reducing the percentage of caseinogen present, the coagulum will immediately become soft; under such conditions the author has found that the curd can be easily and readily split up by the addition of such cereals as are found in Nestle's Food. This food is not a predigested food; but is one which, when prepared for use, immediately exerts upon itself that power of digestion which has been carefully preserved in its constituents. The malt, rich in diastase, acting in conjunction with the pancreatin secretions, digests the fats and albuminoids of the milk, converting the starch of the cereals into sugar (maltose) and neutralizing the tendency of the acids of the stomach to form curds, thus rendering the food of infinite value to the infant, supplying the necessary nutrition and material to strengthen up and build up the human economy, at the same time relieving the stomach of the infant from the expenditure of energy necessary to the digestion of ordinary food.

In feeding babies we have to take the hard-curding milk, which in cow's milk, and feed it to a baby that is used to soft-curding milk from the mother, and that is where the great trouble arises. The hard, lumpy masses of casein that are thrown down by the rennet and acid in the stomach are difficult for the baby to digest, and it is well to bear in mind that digestive enzymes act by contact alone. The casein of cow's milk clots in hard, lumpy masses, the digestive enzymes of the stomach can not get at it, and any means by which we can break up the clot and make it more flocculent will increase the digestibility of the milk. This is the peculiar characteristic that renders Nestle's Food so similar to mother's milk, that being alkaline the curd clots are broken up and the digestive enzymes are permitted to come in direct contact with the coagulum or casein.

Careful analyses made by the leading chemists of England, Germany and America have proved that Nestle's Food in its composition shows a very close resemblance to mother's milk, and that it contains all the elements of a complete nutrition, in a most assimilable form. This food is manufactured from choice milk from healthy pasture-fed cows. The milk is concentrated in vacuo, at low temperature, so as to preserve its original valuable qualities unchanged. To this concentrated milk is added sugar and the soluble elements of wheat flour, in such proportions as most nearly corresponds to the amount of fat, sugar, proteids and the salts of mother's milk. The wheat flour is previously submitted to a special process of baking, by which the insoluble portions are modified and prepared for easy assimilation. The product obtained in this way acts on the casein and prevents the milk from curdling in large lumps.

